# 2-D08 treatment regulates C2C12 myoblast proliferation and differentiation via the Erk1/2 and proteasome signaling pathways

**DOI:** 10.1007/s10974-021-09605-x

**Published:** 2021-06-17

**Authors:** Hyunju Liu, Su-Mi Lee, Hosouk Joung

**Affiliations:** 1 Department of Obstetrics and Gynecology, Chosun University College of Medicine, Gwangju, Republic of Korea; 2 Research Institute of Medical Sciences, Chonnam National University Medical School, Hwasun, Republic of Korea; 3 Department of Internal Medicine, Division of Gastroenterology and Hepatology, Chonnam National University Medical School,, 42, Jebong-ro, Dong-gu, Gwangju, 61469 Republic of Korea

**Keywords:** Myogenesis, Skeletal Muscle, C2C12, Differentiation, 2-D08, SUMOylation

## Abstract

SUMOylation is one of the post-translational modifications that involves the covalent attachment of the small ubiquitin-like modifier (SUMO) to the substrate. SUMOylation regulates multiple biological processes, including myoblast proliferation, differentiation, and apoptosis. 2-D08 is a synthetically available flavone, which acts as a potent cell-permeable SUMOylation inhibitor. Its mechanism of action involves preventing the transfer of SUMO from the E2 thioester to the substrate without influencing SUMO-activating enzyme E1 (SAE-1/2) or E2 Ubc9-SUMO thioester formation. However, both the effects and mechanisms of 2-D08 on C2C12 myoblast cells remain unclear. In the present study, we found that treatment with 2-D08 inhibits C2C12 cell proliferation and differentiation. We confirmed that 2-D08 significantly hampers the viability of C2C12 cells. Additionally, it inhibited myogenic differentiation, decreasing myosin heavy chain (MHC), MyoD, and myogenin expression. Furthermore, we confirmed that 2-D08-mediated anti-myogenic effects impair myoblast differentiation and myotube formation, reducing the number of MHC-positive C2C12 cells. In addition, we found that 2-D08 induces the activation of ErK1/2 and the degradation of MyoD and myogenin in C2C12 cells. Taken together, these results indicated that 2-D08 treatment results in the deregulated proliferation and differentiation of myoblasts. However, further research is needed to investigate the long-term effects of 2-D08 on skeletal muscles.

**Supplementary Information:**

The online version contains supplementary material available at 10.1007/s10974-021-09605-x.

## Introduction

The study of myogenesis is crucial in understanding the cellular and molecular regulation of the multi-stage process of muscle formation. Skeletal muscle plays a significant role in physical activity and in regulating energy production and the balance of body protein mass. Myoblast proliferation, differentiation, and formation of multinucleated myofibers are multiple cellular events constituting skeletal muscle development (Buckingham and Rigby 2014; Chal and Pourquie 2017; Dias et al. 1994). The myogenic process is regulated by several extracellular molecules and certain critical signaling pathways such as p38MAPK, ERK1/2, and PI3K/AKT (Jiang et al. 1998, 1999; Weston et al. 2003; Yang et al. 2006). They in turn lead to the subsequent activation of myogenic regulatory factors (MRFs), including MyoD, myogenin, and MRF4, that modulate muscle-specific gene expression (Buckingham and Rigby 2014; Ganassi et al. 2018; Mal and Harter 2003; Sartorelli and Caretti 2005; Tapscott 2005).

Among the signaling pathways involved, the extracellular signal-regulated kinase 1/2 (ERK1/2) pathway plays important roles in myoblast differentiation (Boyer et al. 2019; Oishi et al. 2019; Yang et al. 2006). This pathway, as well as members of the mitogen-activated protein kinase (MAPK) pathway, is involved in regulating numerous cellular processes such as cell growth, differentiation, apoptosis, and necrosis (Cagnol and Chambard 2010; Locatelli et al. 2016; Schevzov et al. 2015; Tasaki et al. 2011; Zou et al. 2019). Previous studies indicated that ERK1/2 is required for the proliferation and differentiation of myoblast cells (Michailovici et al. 2014; Wu et al. 2000; Yang et al. 2006). The activated ERK1/2 signaling promotes skeletal muscle cell proliferation but negatively regulates myogenic differentiation (Jones et al. 2001). There is some evidence that activated ERK1/2 signaling modulates nuclear factor of activated T cells c1(NFATc1) in the regulation of cell proliferation, apoptosis, and differentiation in myoblast cells (Chen et al. 2017; Cicek et al. 2011; Robbs et al. 2013).

In addition, the balance of muscle (protein) mass is regulated by the relationship between muscle protein synthesis and muscle protein breakdown (Kumar et al. 2009; Millward et al. 1976; Phillips et al. 1997). It is necessary to understand the regulation of muscle protein synthesis and breakdown at the molecular level (Anthony 2016; Bonaldo and Sandri 2013; Schiaffino et al. 2013). In particular, All tissues contain multiple proteolytic systems for protein breakdown, including liver, cardiac, and skeletal muscle (Fagan et al. 1987; Lyon et al. 2013; Zaouali et al. 2017). It is clear now that most intracellular proteins are degraded by the Ub (ubiquitin)-proteasome pathway (Fagan et al. 1987; Kitajima et al. 2020; Lecker et al. 2006). The Ub-proteasome pathway is believed to degrade key skeletal muscle proteins and is the primary regulatory mechanism in muscle atrophy (Attaix et al. 2005; Kitajima et al. 2020; Milan et al. 2015).

2-D08(2′,3′,4′-trihydroxyflavone) is mechanistically a cell-permeable inhibitor of protein SUMOylation (Kim et al. 2014). 2-D08 is specific inhibitor of SUMOylation that blocks the transfer of SUMO (small ubiquitin-like modifier) from the E2 (Ubc9) thioester conjugate to substrates (Kim et al. 2014). According to previous studies, 2-D08 may offer a novel anti-aggregative and neuroprotective effect against amyloid beta protein neurotoxicity in Alzheimer’s disease (Marsh et al. 2017). 2-D08 inhibits the migration of pancreatic cancer cells and induces K-Ras deSUMOylation (Choi et al. 2018). Furthermore, 2-D08-induced ROS accumulation mediates cell apoptosis in acute myeloid leukemia (AML) cells, possibly through deSUMOylation of NOX2 (Zhou et al. 2019). However, the individual role of 2-D08 in treating skeletal myoblast cells remains unclear.

Herein, we showed that 2-D08 suppresses cell viability in C2C12 cells. Furthermore, during the differentiation stage, 2-D08 inhibits myogenic differentiation by downregulating the expression of MyoD, myogenin, and myosin heavy chain (MHC), as well as by decreasing the rate of myotube formation. In addition, 2-D08 treatment rescued Erk1/2 activation and regulated MyoD and myogenin expression via the 26 S proteasome pathway.

## Materials and methods

### Cell culture and treatments

C2C12 cells were purchased from ATCC (American Type Culture Collection, VA, USA) and cultured with high-glucose Dulbecco’s Modified Eagle’s Medium (DMEM, 11995-065, Gibco) containing 15% fetal bovine serum (SH30919.03, Hyclone) and 1% penicillin-streptomycin (15140122, Gibco) at 37 °C and under 5% CO_2_. To induce the differentiation of C2C12 cells, the growth medium (GM) was replaced with 2% horse serum (HS, 16050-130, Gibco), containing DMEM and 1% antibiotics. The chemical 2-D08 was purchased from Merck Millipore (SML1052-5MG). 2-D08 was diluted to the final concentrations indicated in the Figures and C2C12 cells were incubated with the compound for the time indicated before harvesting. The proteasome inhibitor MG132 was purchased from Selleckchem (S2619). C2C12 cells were treated with MG132 at a final concentration of 25 µM in differentiation medium (DM) for 6 h. Dimethyl sulfoxide (DMSO, DMS555.500, BioShop) was used as a control.

### Cell viability assay

To measure cell viability, the MTT assay was used to measure the cell proliferation rate. In brief, C2C12 cells were plated in 96-well plates (5 $\times$ 10^3^ cells/well) overnight under conducive growth conditions and treated with the indicated concentration of 2-D08 for 24 h. MTT solution (M2128, Sigma-Aldrich) was added to each well, and the cells were incubated for 3 h at 37 °C. After removing the medium, 150 µL of DMSO was added to all the wells. The absorbance values were measured at 570 nm using the SpectraMax plus instrument (Molecular Devices).

### Protein preparation and western blot analysis

Total cellular protein lysates were prepared and western blot analyses were performed as previously described (Joung et al. 2014, 2018). C2C12 cells were lysed in an RIPA buffer (R2002, Biosesang) supplemented with protease inhibitors (P8340, Sigma-Aldrich) and the protein concentration of the lysate was determined using the Pierce BCA Protein Assay Kit (#23225, ThermoFisher Scientific). These lysates were denatured by heat treatment, 10% sodium dodecyl sulfate-polyacrylamide gel electrophoresis (SDS-PAGE) was performed to separate the proteins. Next, the separated proteins were electrophoretically transferred onto PVDF membranes (IPVH00010, Merck Millipore), which were then blocked with 5% skimmed milk (262100, BD Difco) and stained with antibodies specific to the target proteins. The primary antibodies used in the present study are as follows: Myosin heavy chain (MHC, MF20), Myogenin (F5D) (Developmental Studies Hybridoma Bank), MyoD (sc-304), SUMO1 (sc-5308) (Santa Cruz Biotechnology), phospho-Erk1/2 (Thr202/Tyr204; 9106), Erk1/2 (4695), phospho-Akt (Ser473; 9271 S), Akt (9272 S), and β-Actin (4967 S) (Cell Signaling Technology). The generated signals were detected using the Immobilon ECL Ultra Western HRP Substrate (WBKLS0100, Merck Millipore) and quantified with a UVITEC Chemiluminescence Imaging System (Alliance Q9 Micro) after hybridization with a horseradish peroxidase (HRP)-conjugated secondary antibody (7074 or 7076 S, Cell Signaling Technology). Specific protein bands detected by western blots were quantified with Q9-Alliance (Uvitec, v18.10) software.

### Immunocytochemistry

Immunostaining for MHC expression was performed as described previously (Joung et al. 2014, 2018). C2C12 cells were fixed with 4% paraformaldehyde for 10 min and permeabilized with 0.2% Triton X-100 (T8787-50ML, Sigma-Aldrich) for 5 min at room temperature. Cells were then blocked with 1% bovine serum albumin (BSA, A7888-50G, Sigma-Aldrich) in phosphate-buffered saline (PBS, LB 001-02, Welgene) and incubated with anti-MHC (MF20) antibody at 1:100 dilution at 4 °C overnight. The anti-MHC antibody was detected by incubating the cells with anti-mouse IgG antibody (Fab2 Alexa Fluor 488 Conjugate, 4408 S, Cell Signaling) at 1:500 dilution for 1 h. The cell nuclei were stained and the slides were mounted using ProLong Gold Antifade Mountant with DAPI (P36935, Invitrogen). Fluorescence was detected using the EVOS FL Cell Imaging System (ThermoFisher Scientific).

### Fusion index

The number of nuclei was calculated using the NIH ImageJ software (version 1.53a). Fusion index was calculated as the number of nuclei inside each myotube divided by the total number of nuclei counted.

### Statistical analysis

The experiments were carried out independently at least thrice. The student’s *t-*test was used to analyze the significance of the difference between two groups. *p < 0.05 and **p < 0.01 were deemed statistically significant. These tests were performed using GraphPad Prism 6 software. All data were expressed as means $\pm$ standard error of the means (SEM).

## Results

### 2-D08 inhibited C2C12 cell viability in a dose-dependent manner

**Fig. 1:**
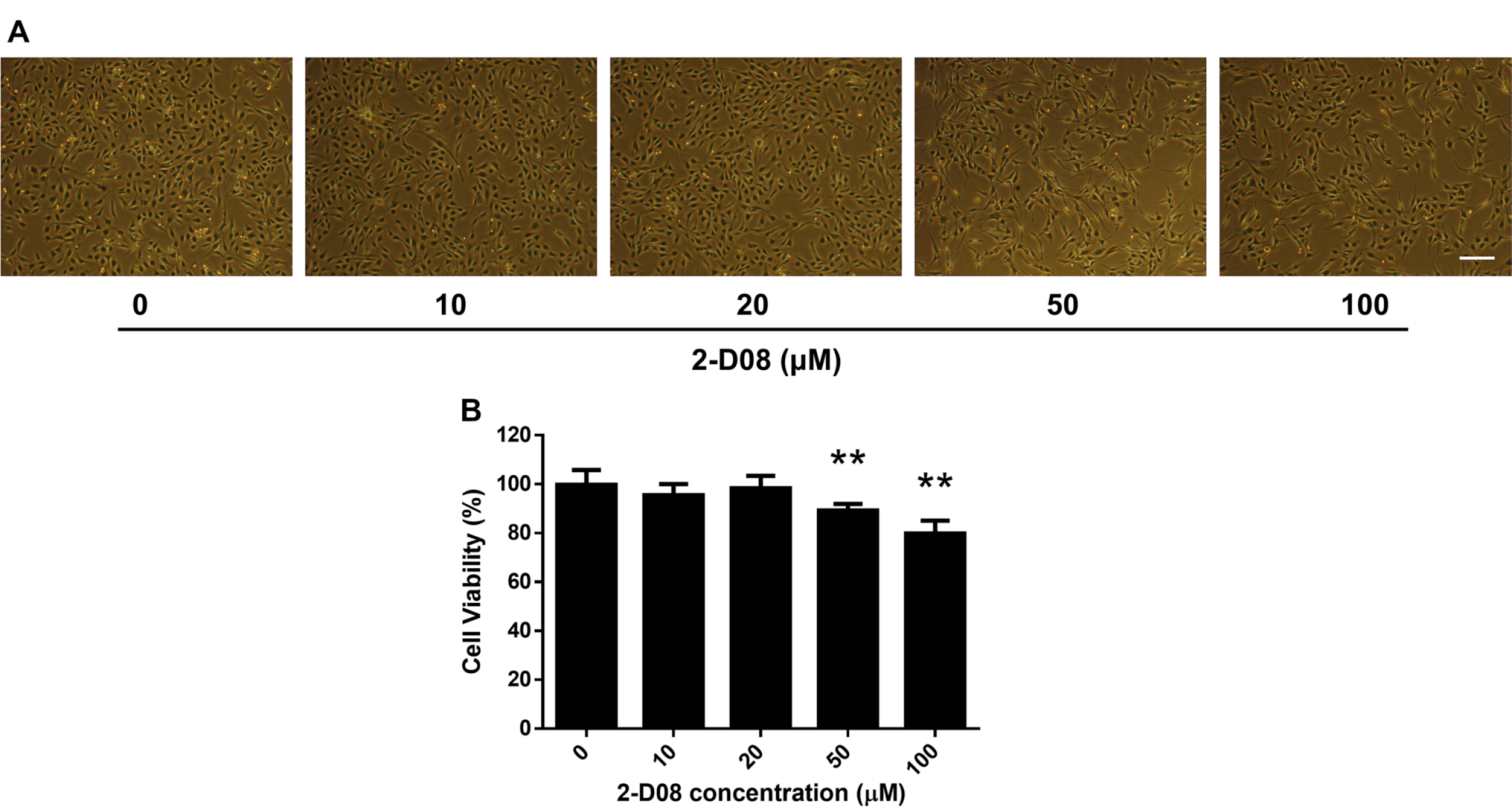
2-D08 impairs C2C12 cell viability. **a** Morphology of untreated C2C12 cells and cells treated with 10, 20, 50, and 100 µM of 2-D08 for 24 h. C2C12 cells were treated with DMSO or 2-D08 at the indicated dose in culture medium. **b** Cell viability of C2C12 cells was measured by the MTT assay as described in Materials and Methods. 2-D08 treatment with 0–100 µM concentration for 24 h. *p < 0.05 and **p < 0.01 vs. the DMSO-treated group. White scale bar = 450 μm. *DMSO* dimethyl sulfoxide

We investigated the cytotoxic effect of 2-D08 treatment on skeletal myogenesis using the well-established C2C12 myoblast cell model (Yaffe and Saxel 1977). To evaluate the role of 2-D08 in C2C12 cells, the cells were incubated with different concentrations of 2-D08 (10–100 µM) for 24-h, and the MTT assay was then performed as described. As shown in Fig. 1a, cell morphology was distinctively observed in the concentration range 50–100 µM compared to the cell death rate observed in control cells after 24-h incubation. Conversely, at the lower concentrations of 10 and 20 µM, the cell morphology was not affected compared to the cell morphology observed in untreated cells. The maximum reduction in cell viability (20%) was observed at the highest dose of 100 µM (*p* < 0.01 versus control) and a dose-dependent decrease in cell viability was observed (Fig. 1b). These data demonstrated that 2-D08 treatment significantly affects C2C12 cell viability.

### 2-D08 inhibited C2C12 myoblast differentiation

**Fig. 2:**
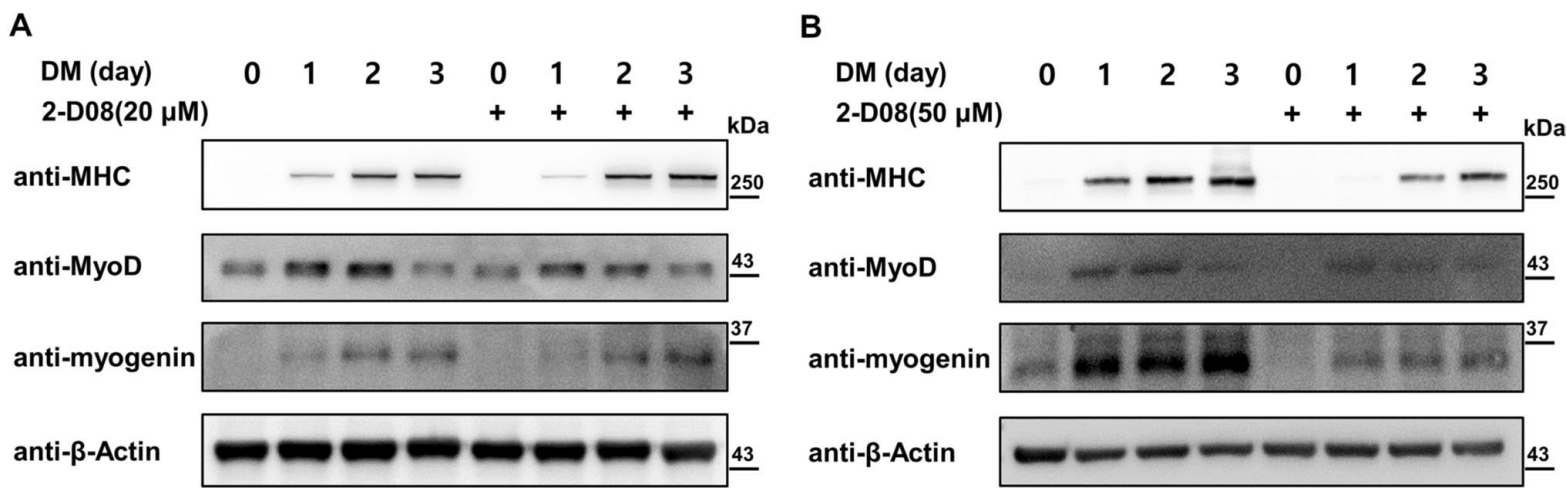
2-D08 regulates myogenic differentiation in C2C12 cells. **a**, **b** C2C12 cells were treated with the indicated amounts (20 and 50 µM) of 2-D08 or DMSO and were induced to differentiate for 3 days. Lysates were processed for western blotting with antibodies against MHC, MyoD, and myogenin, in addition to β-Actin as a loading control. DM, differentiation medium; DMSO, dimethyl sulfoxide; MHC, myosin heavy chain

**Fig. 3:**
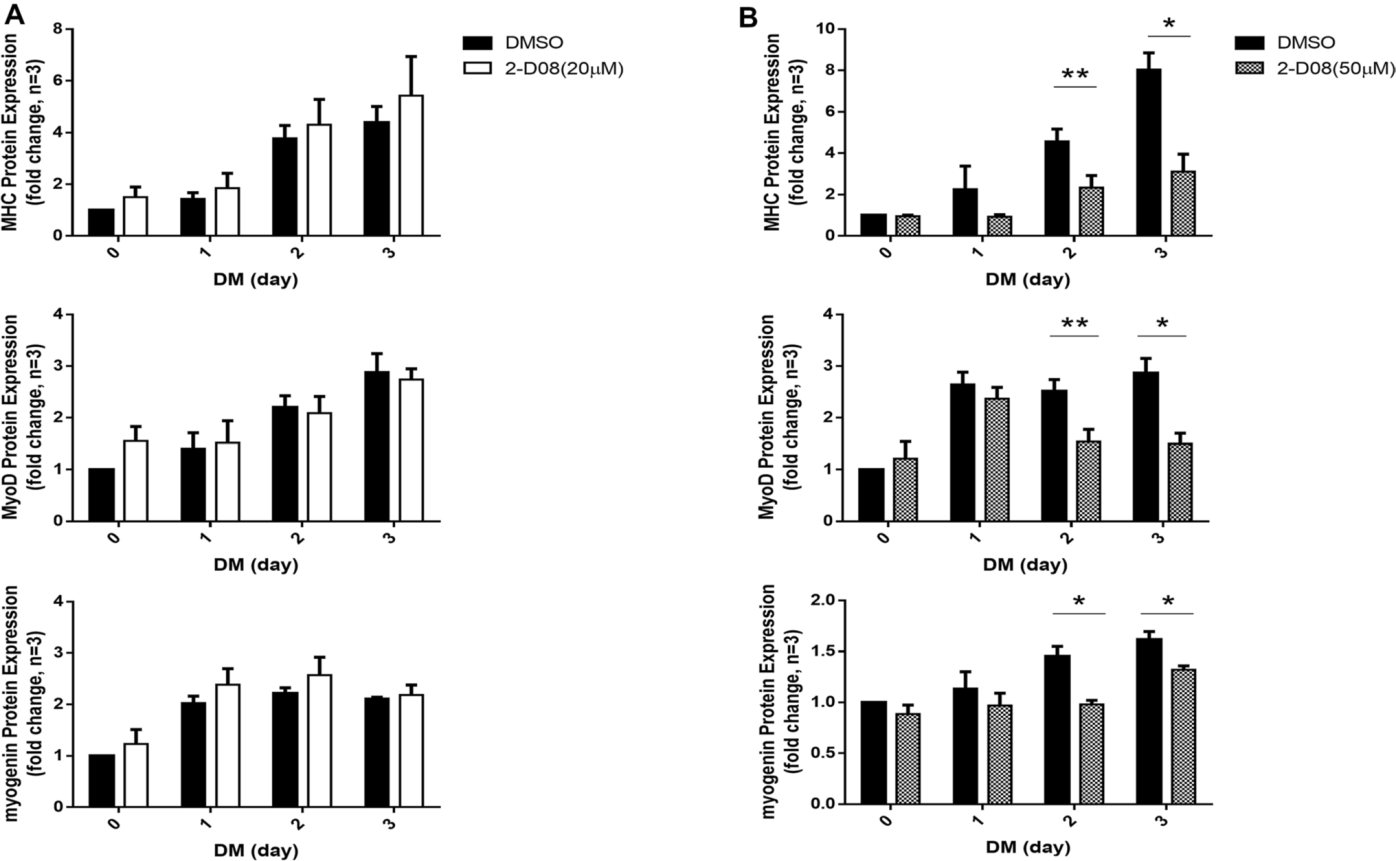
Western blot quantification of MHC, MyoD and myogenin protein levels. **a**, **b** Bar graphs showing the relative MHC, MyoD and myogenin protein levels following treatment of 2-D08 (20 and 50 µM) in DM for 3 days. Relative protein levels were analyzed by quantification of the density of the protein bands with Q9-Alliance software. *p < 0.05 and **p < 0.01 vs. the DMSO-treated group. These experiments were repeated three times. DM, differentiation medium; DMSO, dimethyl sulfoxide; MHC, myosin heavy chain

To examine the effect of 2-D08 on myogenic differentiation, C2C12 cells were incubated with different concentrations of 2-D08 (10–100 µM) and western blot analysis was performed following treatment. Replacement of GM with DM over a 3-day period significantly enhanced the expression of myogenesis marker genes, such as MHC and myogenin (Miller 1990). As per our observation, myotube formation began on days 1–2 upon the addition of DM, and differentiated myotubes were observed on days 2–3. For cellular differentiation, C2C12 cells were treated with/without 2-D08 over 3 days. 2-D08-treated C2C12 cells exhibited decreased MHC expression in a dose-dependent manner compared with corresponding expression level in the control DMSO-treated cells (Supplementary Fig. 1a). Furthermore, we identified a decrease in the levels of Sumo1-conjugated proteins in the presence of a high dose of 2-D08 (Supplementary Fig. 1b). To further investigate the effect of 2-D08 on C2C12 myogenic differentiation, C2C12 cells were induced to differentiate for 3 days in the presence of two different concentrations of 2-D08 (20 µM and 50 µM). Incubation with a low concentration of 2-D08 (20 µM) did not result in an obvious difference in MHC, MyoD, and myogenin expression (Figs. 2a and 3a). However, high concentrations of 2-D08 (50 µM) significantly delayed the protein expression of MHC, MyoD, and myogenin after 48 h compared to the corresponding expression level in the untreated control (*p* < 0.05 and *p* < 0.01, Figs. 2b and 3b). The results indicated that 2-D08 treatment hinders the differentiation of C2C12 cells.

### 2-D08 treatment impaired myoblast fusion

**Fig. 4:**
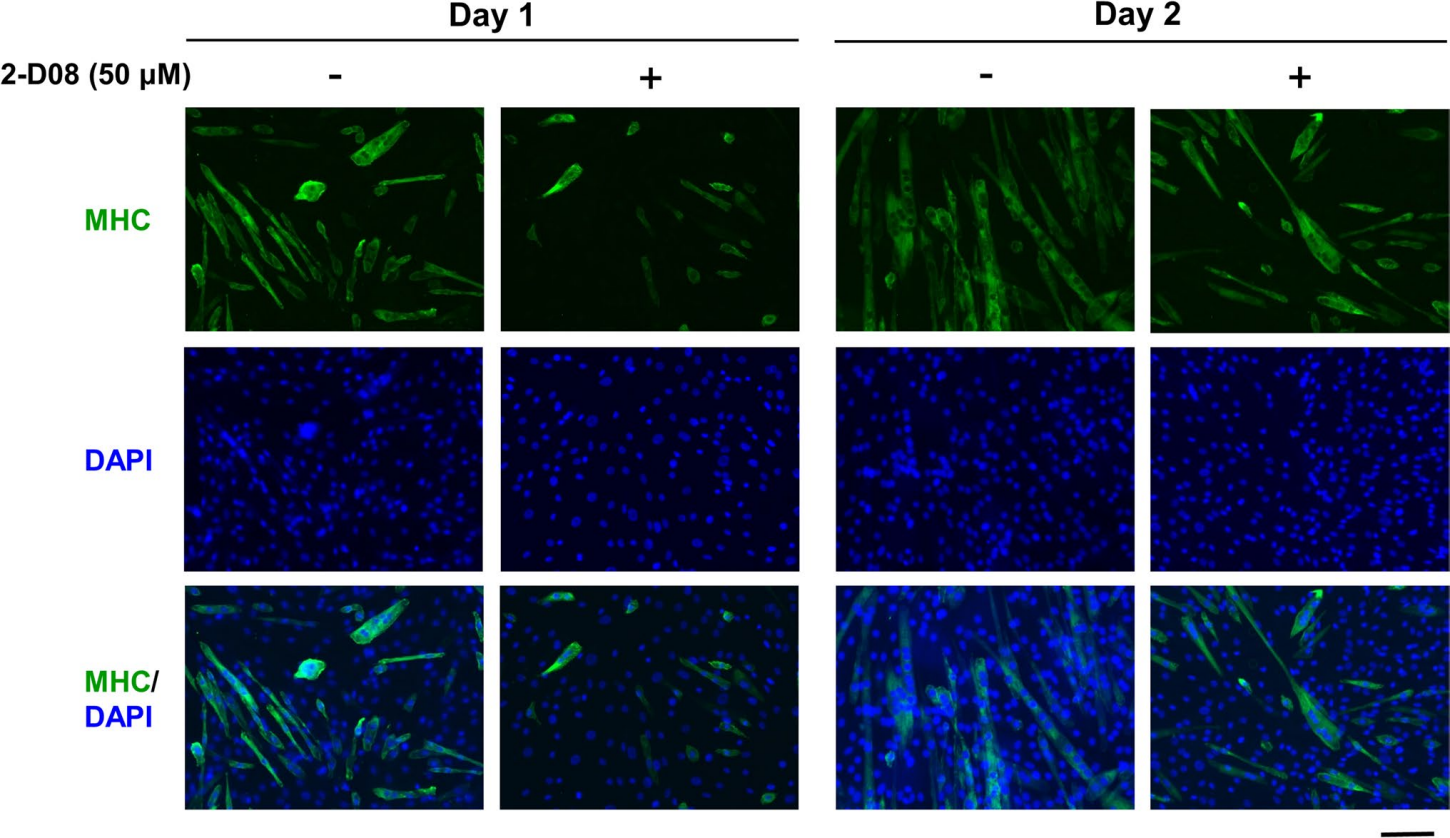
2-D08 inhibits myotube formation in C2C12 cells. To observe myotube formation, fluorescence images of DAPI- and MHC antibody-stained myotubes of C2C12 cells differentiated at 24 and 48 h following 2-D08 treatment (50 µM). Myotube morphology was visualized using MHC (green). Cell nuclei were visualized by DAPI staining (blue). Black scale bar = 100 μm. MHC, myosin heavy chain; DMSO, dimethyl sulfoxide

**Fig. 5:**
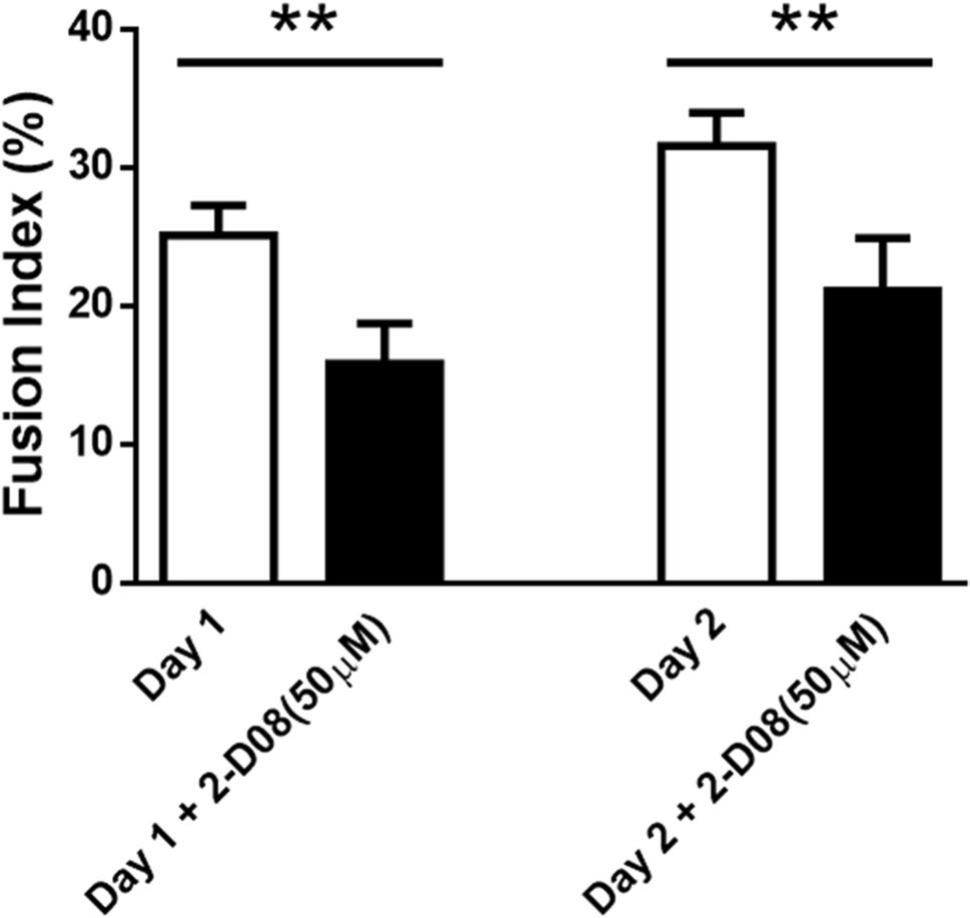
Quantification analysis of results for fusion index for control and 2-D08 treatment group (Fig. 4). Fusion index decreased significantly for the 2-D08 group compared to the control. *p < 0.05 and **p < 0.01 vs. the DMSO-treated group. *DM* differentiation medium

To determine whether treatment with 2-D08 impeded myotube formation, DMSO- and 2-D08-treated C2C12 cells were induced to differentiate for 24 or 48 h, and then immunostained with anti-MHC antibody followed by DAPI staining. The final concentration of 2-D08 was 50 µM because this concentration significantly delayed MHC expression compared to the corresponding expression level in the untreated control (Day 2: *p* < 0.01; Day 3: *p* < 0.05, Figs. 2b and 3b). As shown in Figs. 2, 4-D08-treated C2C12 cells, for 24 or 48 h, delayed the onset of myotube formation and reduced the differentiation of C2C12 compared to control cells. Furthermore, we observed that the addition of 2-D08 for 72 h to the DM resulted in dramatic changes in cell shape, compared to the case for the untreated cells (Supplementary Fig. 2). The quantitative data (fusion index) also showed that treatment with 2-D08 reduced the percentage of MHC-positive cells 24 or 48 h after C2C12 differentiation (Fig. 5). Treatment with 2-D08 (50 µM) resulted in a 35 ~ 38% decrease of the fusion index compared to controls on day 1–2 of differentiation (p < 0.01). The results presented above indicated that 2-D08-treated C2C12 cells result in significant inhibition of myogenic differentiation, as evidenced by reduced numbers of MHC-positive myotubes compared to the myotubes observed in the control.

### 2-D08 found to mediate the Erk1/2 signaling pathway in C2C12 cells

**Fig. 6:**
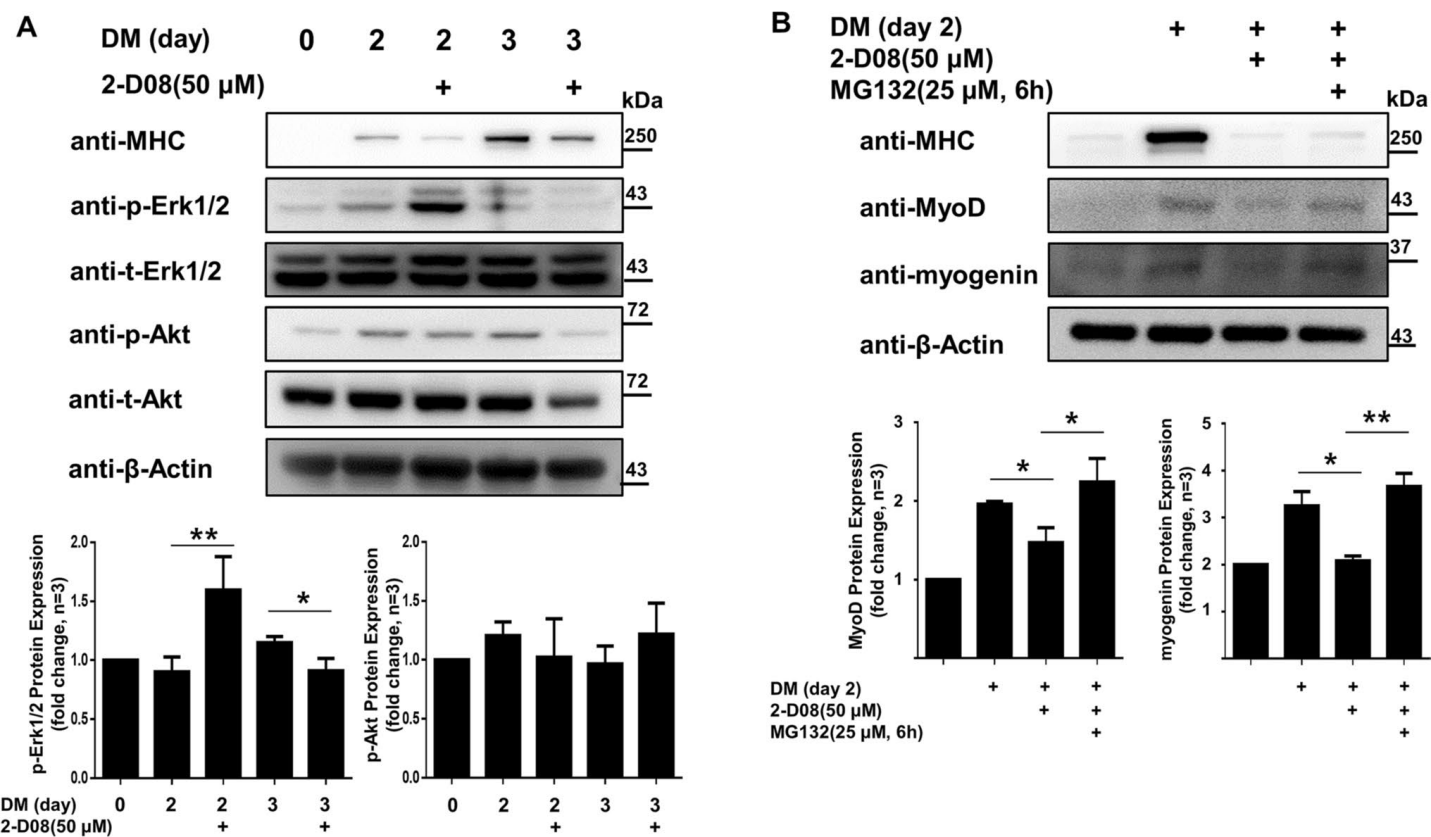
2-D08 suppresses C2C12 cell differentiation via Erk 1/2 signaling and the proteasome pathway. **a** C2C12 cells treated with 50 µM of 2-D08 in DM. Treated cells were lysed and analyzed using western blots with the respective antibodies against phosopho-Erk1/2 (Thr202/Tyr204), Erk1/2, phospho-Akt (Ser473), and Akt, in addition to β-Actin as a loading control (upper panel). The intensity of blots was quantified by densitometry using Q9-Alliance software (lower panel). **b** C2C12 cells were pretreated with/without 2-D08 (50 µM) for 48 h under differentiation conditions and then pretreated with/without MG132 (25 µM) for 6 h. The cell lysates were immunoblotted with the antibodies against MHC, MyoD, and myogenin, in addition to β-Actin as a loading control (upper panel). The intensity of blots was quantified by densitometry Q9-Alliance software (lower panel). *p < 0.05 and **p < 0.01, compared to the indicated experimental group (bars). These experiments were repeated three times. DM, differentiation medium; p-Erk1/2, phosphorylated Erk1/2(Thr202/Tyr204); t-Erk1/2, total Erk1/2; p-Akt, phosphorylated Akt (Ser473); t-Akt, total Akt

To analyze the molecular mechanisms of 2-D08 in C2C12 cells, we also evaluated the time course of changes in Erk1/2 and Akt phosphorylation after treatment with differentiation culture. As previously described, the Erk1/2 and Akt pathways have been implicated in the control of muscle differentiation (Boyer et al. 2019; Jiang et al. 1999; Jones et al. 2001). After treatment with 50 µM 2-D08 in DM for 3 d, 2-D08-treated C2C12 cells exhibited a strongly elevated level of active phospho-Erk1/2 (Thr202/Tyr204) on day 2 compared with the corresponding level in vehicle-treated cells (*p* < 0.01); however, there was a return to the basal level on day 3 (*p* < 0.05) (Fig. 6a). In contrast, there were no obvious differences in the Akt phosphorylation (Ser473) level on day 2, whereas 2-D08 decreased both the phospho-Akt (Ser473) and Akt expression levels on day 3 (Fig. 6a). Our data indicated that the anti-myogenic effects of 2-D08 are dependent on the activation of the Erk1/2 pathway.

### 2-D08 promoted proteasome-mediated degradation of myogenic regulatory factors

We also investigated whether 2-D08 treatment promoted MyoD and myogenin degradation via the proteasome pathway. MG132 has been widely used in proteasome inhibition research (Kisselev et al. 2012; Lee and Goldberg 1998). To test our hypothesis, we treated differentiated C2C12 cells with MG132 (25 µM) or vehicle for 6 h. As observed in a previous experiment, 2-D08 also downregulated the protein expression of MHC, MyoD, and myogenin. As shown in Fig. 6b, the amount of MyoD and myogenin was significantly lower in 2-D08-treated differentiated C2C12 cells than in untreated cells (*p* < 0.05). In contrast, both MyoD and myogenin protein levels were significantly rescued in 2-D08-treated C2C12 cells treated with MG132 compared to MG132-untreated cells (MyoD: *p* < 0.05; myogenin: *p* < 0.01). However, MHC expression was downregulated in MG132-treated cells in contrast with the MHC expression seen in untreated cells; furthermore, no difference was observed between the MG132-treated and untreated groups. Accordingly, our results also revealed that the inhibition of the proteasome pathway can partially prevent MyoD and myogenin degradation.

## Discussion

In the present study, we determined that 2-D08 treatment in C2C12 cells decreased cell viability in a dose-dependent manner. We found that continued treatment with 2-D08 (20–50 µM) significantly downregulated MHC, MyoD, and myogenin expression, in addition to significantly delaying myotube formation (fusion index). We also found that 2-D08 inhibits C2C12 cell differentiation via the Erk 1/2 signaling and proteasome pathway.

2-D08, a SUMO E2 inhibitor, has been employed as a SUMOylation inhibitor (Kim et al. 2014), and various biological properties are ascribed to 2-D08 in vitro*.* A previous study revealed that 2-D08 may exert a novel anti-aggregatory and neuroprotective effect against amyloid beta protein neurotoxicity in Alzheimer’s disease (Marsh et al. 2017). Choi et al. (2018) indicated that 2-D08 inhibited cell migration and invasion by mediating K-Ras deSUMOylation in pancreatic cancer cells. It was reported that 2-D08 induced apoptosis of the human AML cell line through ROS accumulation, in which Nox2 deSUMOylation may play an important role (Zhou et al. 2019). These results implied that the function of 2-D08 has generated considerable interest and its potential effects are promising. In particular, its antitumor effects have attracted a great deal of attention (Choi et al. 2018). However, our results showed that 2-D08 treatment generates a negative effect on C2C12 cell proliferation and differentiation. Although we only used mouse myoblast-derived C2C12 cells, 2-D08 can cause toxicity and result in muscle damage.

As described previously, we showed that 2-D08 impairs C2C12 cell differentiation by repressing the expression of MyoD, myogenin, and MHC while delaying myotube formation. This finding is similar to the results of a previous study (Riquelme et al. 2006) in which the knockdown of Ubc9 (SUMO E2-conjugating enzyme) decreased myotube formation in C2C12 cells without affecting MyoD and myogenin expression. These results suggest that 2-D08 may be associated with other pathways in C2C12 cells, compared to Ubc9 knockdown. Our experiments further confirmed that the suppression of proliferation of 2-D08-treated C2C12 cells could have resulted in fewer C2C12 cells that are available to differentiate at a high concentration (~ 50 µM) (Fig. 5). The detailed underlying mechanisms are unclear; however, our results may aid in explaining that the observed repression of proliferation could result in an insufficient number of cells required for differentiation.

It is interesting that our data show that 2-D08-treated C2C12 cells manifested an increased level of phosphorylated Erk1/2 (Thr202/Tyr204) within 48 h of treatment, whereas the phosphorylation level decreased subsequently. The ERK1/2 signaling pathways play an important role in cell proliferation, differentiation, and apoptosis. In addition, it has been reported that ERK1/2 activity is associated with decreased cell proliferation (Cagnol and Chambard 2010; Jones et al. 2001). Thus, we assumed from the result of the MTT assay (Fig. 1b) that the ERK 1/2 pathway may be linked to 2-D08-treated C2C12 cell proliferation.

Moreover, most proteolysis mediated by the proteasome system must undergo ubiquitination before degradation, following which E3 ubiquitin ligases play essential roles in numerous cellular processes (Clague and Urbe 2010; Pickart and Eddins 2004). Muscle-specific E3 ubiquitin ligases (for example, MAFbx/atrogin-1 and MuRF-1) are important players in skeletal muscle atrophy (Attaix et al. 2005; Rom and Reznick 2016). In addition, ERK1/2 activation is also elevated in atrophied and damaged skeletal muscles (Hilder et al. 2003; Kato et al. 2002). An increased phosphorylation level of ERK1/2 is associated with the overexpression of muscle-specific E3 ubiquitin ligases in C2C12 cells (Hemdan et al. 2009). Furthermore, MAFbx and MuRF-1 ubiquitin ligases enhance proteasome degradation of MyoD and myogenin (Jogo et al. 2009; Lagirand-Cantaloube et al. 2009; Tintignac et al. 2005). As shown in Figs. 2, 6-D08 exposure may partially regulate muscle differentiation via regulation of the ERK1/2-mediated proteasome pathway. These results indicated that 2-D08 can even cause muscle atrophy by acting on proteasomal systems.

In conclusion, we identified and characterized the negative effects of the SUMOylation inhibitor 2-D08 and the underlying molecular mechanisms in the myogenesis of C2C12 cells. In brief, 2-D08 significantly hinders myoblast differentiation and the anti-myogenic effect of 2-D08 is mediated through Erk1/2 activation and the proteasome pathway. There are certain limitations to our study, in that all myogenic cells from different lines, such as primary myoblasts or human skeletal myoblast cells, were not evaluated in our analyses. Furthermore, additional in vivo studies are warranted to determine the significance of the present study. Although extensive research is needed to fully understand the biological implications, these results could prove significant in that 2-D08 treatment could lead to the repression of proliferation and impact the myogenic process in C2C12 cells.

## Supplementary Information

Below is the link to the electronic supplementary material.

Supplementary material 1 (DOCX 1444.2 kb)
