# Supplementary material for: 2-D08 treatment regulates C2C12 myoblast proliferation and differentiation via the Erk1/2 and proteasome signaling pathways
Source: J Muscle Res Cell Motil. 2021 Jun 17;42(2):193–202. doi: 10.1007/s10974-021-09605-x (PMC8332585; doi:10.1007/s10974-021-09605-x)
Supplement: Supplementary file 1 — Supplementary material 1 (DOCX 1444.2 kb) [file 10974_2021_9605_MOESM1_ESM.docx]

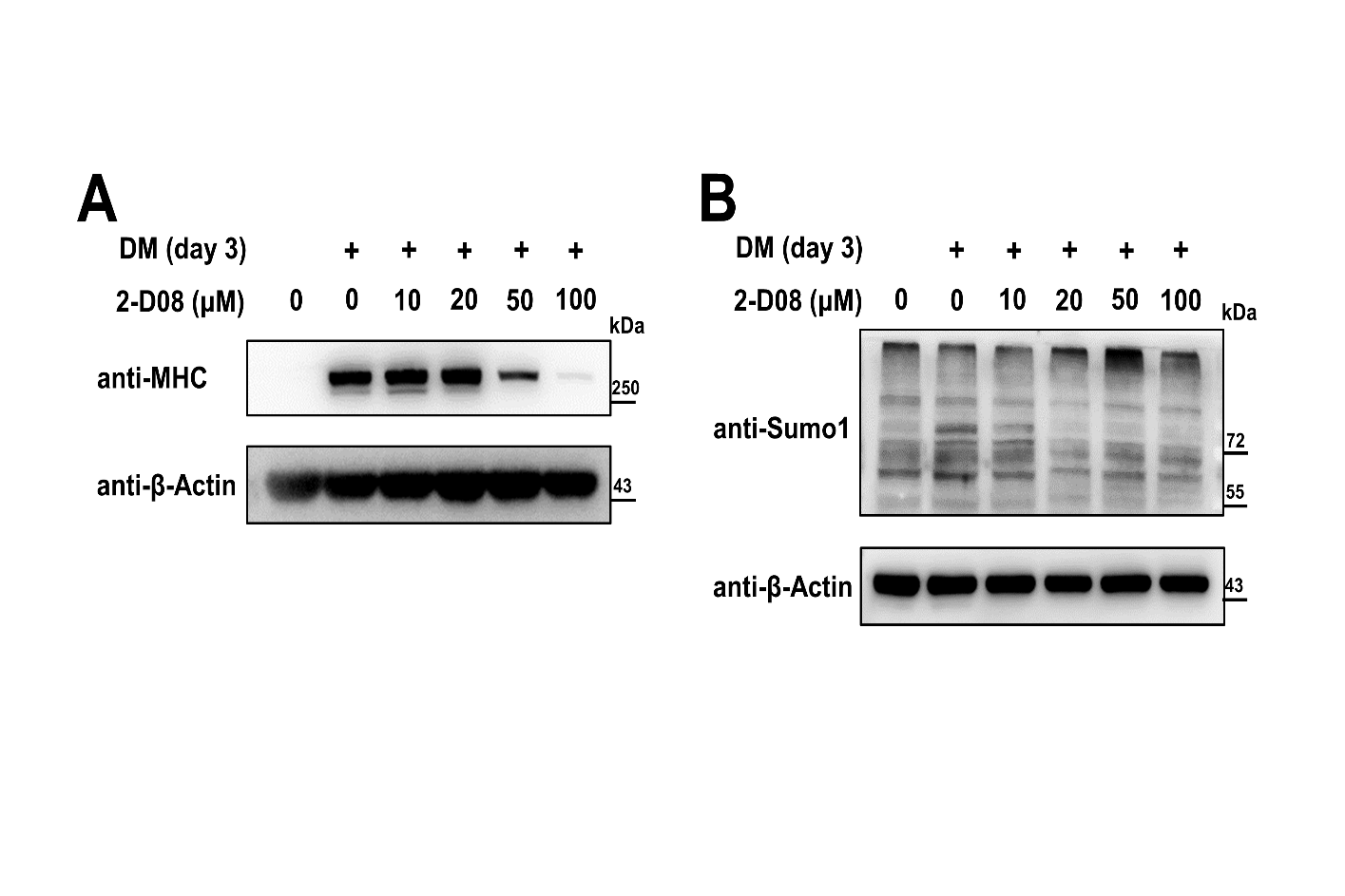


**Supplementary Fig. 1** MHC protein expression in 2-D08-treated C2C12 cells. **a** C2C12 cells were treated with various concentrations (0,10,20,50, and 100 μM) of 2-D08 or DMSO and were induced to differentiate for 3 days. DMSO was used as the control. Lysates were processed for western blotting with antibodies against MHC and β-Actin. DM, differentiation medium. **b** Immunoblotting of Sumo1-conjugated proteins in C2C12 cells treated with 2-D08 at the indicated concentrations for 3 days.


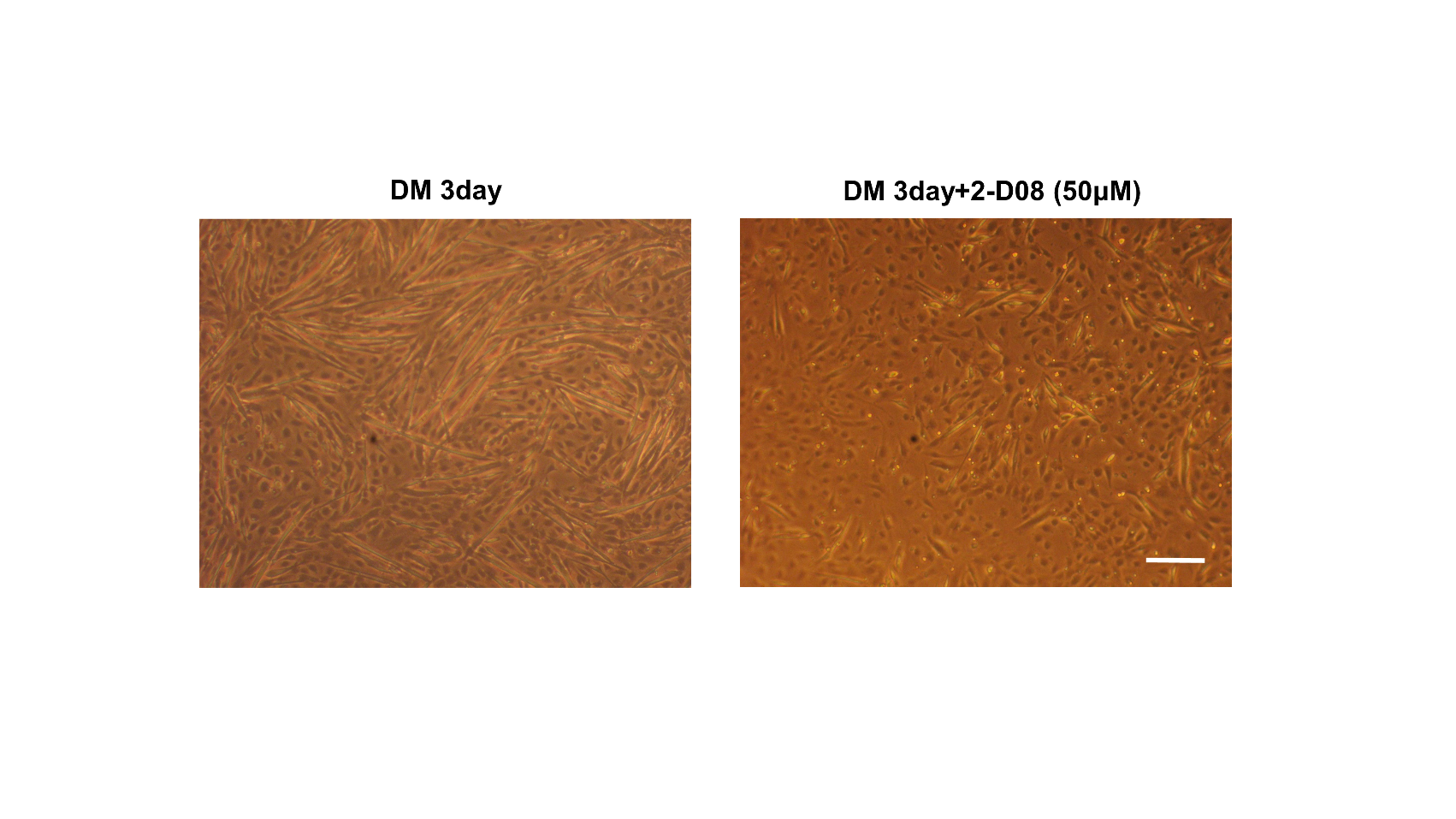


**Supplementary Fig. 2** Effect of 2-D08 (50 μM) on the morphology of C2C12 cells at 72 h. 2-D08 induced morphological change in cells and did not induce the differentiation of C2C12 cells in this study. White scale bar = 450 μm
